# Topological Data Analysis for Unsupervised Feature Selection in Large Scale Spatial Omics Data Sets

**DOI:** 10.1007/s11538-026-01618-2

**Published:** 2026-03-04

**Authors:** James Boyle, Gregory Hamm, Eleanor Williams, Robin JG Hartman, Magnus Söderburg, Ian Henry, Michael Casey

**Affiliations:** 1 Data Science and AI, Translational Science & Experimental Medicine, Research and Early Development, Cardiovascular, Renal and Metabolism, Biopharmaceuticals R&D, AstraZeneca, Cambridge, UK; 2 Mathematical Institute, University of Oxford, Oxford, UK; 3 Integrated Bioanalysis, Clinical Pharmacology and Safety Sciences, Biopharmaceuticals R&D, AstraZeneca, Cambridge, UK; 4 Cambridge Stem Cell Institute, University of Cambridge, Cambridge, UK; 5 Predictive AI and Data, Clinical Pathology and Safety Sciences, Biopharmaceuticals R&D, AstraZeneca, Cambridge, UK; 6 Cardiovascular Renal and Metabolism Pathology, Clinical Pharmacology and Safety Sciences, Biopharmaceuticals R&D, AstraZeneca, Gothenburg, Sweden

**Keywords:** Spatial Transcriptomics, Topological Data Analysis, Persistent Homology, Mass Spectrometry Imaging, Spatially Variable Gene

## Abstract

Spatial transcriptomics studies are becoming increasingly large and commonplace, necessitating simultaneous analysis of a large number of spatially resolved variables. Correspondingly, a diverse range of methodologies have been proposed to compare the spatial expression structure of genes. Here, we apply persistent homology, a method from topological data analysis, to produce a continuous quantification of spatial structure in a given gene’s expression, and show how this can be used for downstream tasks such as spatially variable gene identification. We explore the unique advantages of topology for this task, deriving biologically meaningful insights into kidney disease and myocardial infarction using public spatial transcriptomics data. We also show how the non-parametric nature of homology enables our methodology to extend naturally to other spatial omics modalities, demonstrating this on a spatial metabolomics sample. Our work showcases the advantages of using a continuous quantification of spatial structure over *p*-value based approaches to SVG identification, the potential for developing unified methods for the analysis of different spatial omics modalities, and the utility of persistent homology in big data applications.

**Supplementary Information:**

The online version contains supplementary material available at 10.1007/s11538-026-01618-2.

## Introduction

Spatial transcriptomics experiments measure gene expression in 2-dimensional space, up to the resolution of a supra-cellular well, cell, or subcellular location (Moses and Pachter 2022; Marx 2021). A common task when analysing spatial transcriptomics data is to identify genes that exhibit spatial structure in their expression, commonly referred to as *Spatially Variable Genes* (SVGs) (Charitakis et al. 2023; Svensson et al. 2018).

SVGs are the spatial analogue of Highly Variable Genes in single-cell transcriptomic data. The central task of single-cell transcriptomics analysis is the classification of cells into discrete types, typically done by first identifying those genes with substantially greater variance in expression than expected, which are then more likely to discriminate between different cellular identities.

Analogously, the aim of SVG selection is to identify a subset of genes likely to be indicative of spatial features in spatial transcriptomics data. SVG identification methods are typically based on null hypothesis rejection, with the null assuming no dependence of expression on spatial location (Svensson et al. 2018; Zhu et al. 2021), or through computing a continuous measure of the degree of spatial structure in each gene’s expression (Andersson and Lundeberg 2021). The latter approach provides a more informative quantification of the degree of spatial variability in each gene’s expression, and a continuous measure of spatial structure can enable meaningful comparisons of the degree of spatial organisation in the expression of different genes across tissue samples. Significant differences in spatial structure may highlight disease related features such as breakdown of tissue architecture, expansion of fibrotic regions, or enhanced immune infiltration into previously more organised tissue compartments.

Applications of previously published continuous measures of spatial structure (such as the sepal score (Andersson and Lundeberg 2021)) have not been extended beyond SVG identification within a single sample. For comparisons between samples to be meaningful, the quantification must be robust to the reasonable amounts of noise, tissue deformation, and variations in tissue morphology inherent to spatial transcriptomics experiments. Additionally, the metric should also be able to detect a very broad range of spatial structures. In other words, we wish to quantify the level of spatial structure in gene expression in a way that is minimally sensitive to the specific geometry or coordinate system of the tissue section, whilst being comprehensive as to what counts as spatial structure. These considerations lead very naturally to the mathematical field of topology and the tools of topological data analysis, which aim to provide coordinate-free characterisations of spatial organisation that are preserved under continuous deformations.

We present an approach based on persistent homology (PH) (Otter et al. 2017; Wasserman 2018) that quantifies spatial variability via the topological activity in a gene’s expression pattern (Carlsson 2009). Loosely speaking, our methodology is based on quantifying the number and significance of ‘hotspots’ of expression, taking the presence of one or more distinct regions of differential gene expression as indicative of spatial structure. Via this highly generalised notion of spatial structure, we are able to detect a broad range of spatial patterns in a way that is robust to variance in tissue morphology. Moreover, using topology we are able to avoid restrictive assumptions about the statistical distribution of gene expression or gene count data. This enables our methodology to be naturally extended to other spatial omics modalities, which we demonstrate by analysing a mass spectrometry imaging sample (Buchberger et al. 2018).

We explore the capabilities of persistent homology for SVG identification by using our approach to analyse spatial transcriptomics data from kidney disease and myocardial infarction samples. We also show how our topological quantification of spatial structure can be used to automatically identify genes which show a difference in spatial expression between acute kidney injury and chronic kidney disease samples. We illustrate the generalisability of persistent homology by using our methodology to identify spatially variable metabolites in a spatial metabolomics sample.

In order to assess whether persistent homology brings new capabilities to the task of SVG identification, we also compare results from our method with those obtained using a range of other popular SVG identification techniques. We find that persistent homology detects a broader range of spatial structures, and produces more consistent results across different samples and biological settings. In comparison to Sepal, the only other SVG identification method the authors are aware of that performs SVG identification via a continuous quantification of spatial variability, we find that our topology based score identifies novel patterns of spatial structure, and is more effective for downstream analytical tasks.

Our methodology also has some novelty as an application of persistent homology to the automatic analysis of a large number of spatially resolved variables. Persistent homology has previously been used for analysing spatial structure in spatial transcriptomics data and other biological settings (Rizvi et al. 2017; Rabadán and Blumberg 2019; Benjamin et al. 2022), but as far as the present authors are aware, the present work is more distinct as a “big data" application of persistent homology, in which persistent homology is used to automatically perform some analytical task on a large number of spatially resolved variables, the output of which does not involve any manual inspection of the persistent homology outputs. In other words, our pipeline could be comfortably used by a practitioner without any knowledge of persistent homology or topological data analysis. We thus hope this work highlights not just the utility of persistent homology for analysing full transcriptome spatial transcriptomics data sets, but also the potential of persistent homology for use in other high dimensional settings.

We find our spatial structure score most effective as an unsupervised exploratory tool. That is, given spatial transcriptomics data on a large number of genes, we find that persistent homology effectively triages the data down to a smaller number of genes which, for example, display notable spatial structure, which can then be subjected to further analysis. As such, our methodology provides a geometric complement to existing analysis pipelines, quantifying a property of gene expression that can be used in conjunction with other bioinformatics tools (such as Seurat (Hao et al. 2021) and Scanpy (Wolf et al. 2018)).

The rest of the paper is organised as follows. We first provide a non-technical outline of the process of going from the output of a spatial transcriptomics experiment to a list of SVGs. We then fill in the technical details, highlighting the special considerations needed when applying persistent homology to SVG identification. We then evaluate the capabilities of our spatial structure score for SVG identification by applying our methodology to spatial transcriptomics data from kidney disease and myocardial infarction samples, before showing how our persistent homology score can be used for other analytical tasks, and can be extended to other spatial omics modalities.

## Method Overview

**Fig. 1:**
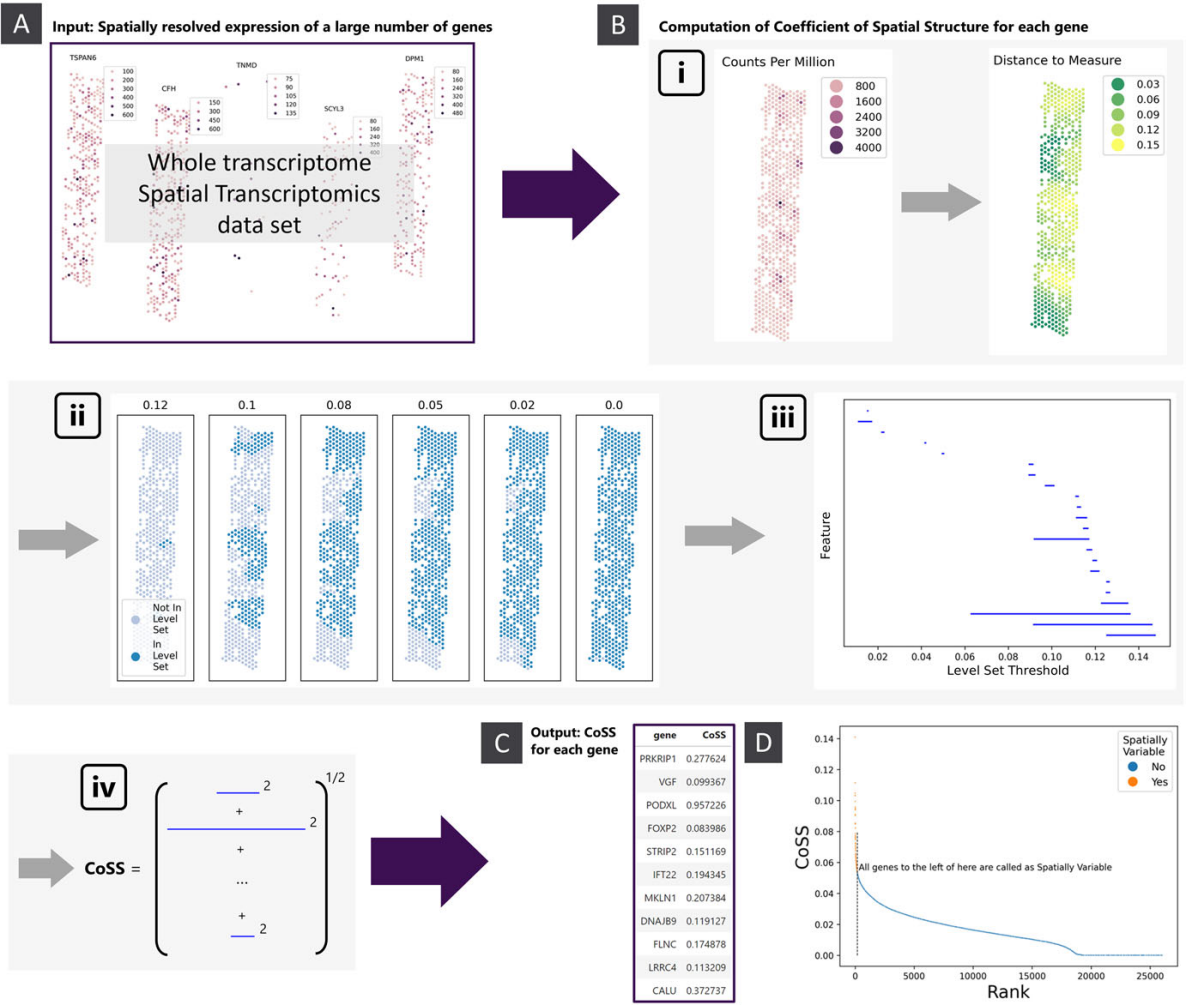
Computation of spatial structure scores. A) The input consists of spatial transcriptomics data on some large number of genes, with expression resolved to a common set of co-ordinates. B) For each gene we compute a single number, the CoSS, quantifying the amount of spatial structure in that gene’s expression: i) Original expression data (in counts per million) and the smoothed expression used for the gene PODXL in an acute kidney infection tissue sample (sample 30-10125 from Lake et al. (2023)). ii) Level sets of the smoothed expression in panel B(i), at various thresholds. iii) Barcode for the 0-dimensional persistent homology of the upper star filtration from the smoothed expression in panel B(i). iv) Computation of the CoSS from the barcode diagram. C) The output consists of the CoSS for each gene in the input data. D) CoSS-rank plot for all genes in the tissue sample in B. The CoSS cutoff for declaring a gene as spatially variable is automatically selected based on the curvature of the CoSS-Rank curve

We take as input the spatially resolved expression of a large number of genes over a fixed set of co-ordinates. For each gene, we use persistent homology to compute a single number, the *Coefficient of Spatial Structure* (CoSS), that quantifies the amount of spatial structure in that gene’s expression pattern (figure 1A,C). In this section we provide a non-technical overview of how we compute the CoSS for a single gene (figure 1). For the full mathematical details, see *Methods*.

Consider the surface plot of the expression of a gene. Genes with a high degree of spatial structure will have well defined regions where their expression is significantly higher or lower than the surrounding tissue, which will appear as distinct hills and valleys in this expression landscape. By contrast, genes with low spatial structure will have a relatively flat, featureless expression landscape. The CoSS is based on this intuition, quantifying spatial structure by examining the number and prominence of tissue regions with substantially higher or lower gene expression than the surrounding tissue.

First we compute a smoothed version of the gene’s expression (figure 1B(i), (Wasserman 2018)). As discussed above for SVGs we would expect the surface plot of this smoothed expression to be more ‘hilly’. We examine the topography of this landscape by considering *level sets* of the smoothed expression, i.e. regions of the underlying tissue where the smoothed expression exceeds a specified threshold (figure 1B(ii)). As we lower this threshold continuously from its maximum value down to zero, we observe how regions of high expression emerge and combine. A region of tissue with substantially higher expression than its surroundings will appear (be ‘born’, in the language of persistent homology) as a distinct component in the level set at a high threshold, and will only merge with the rest of the tissue (‘die’) at a much lower threshold when the surrounding tissue appears in the level set (Wasserman 2018). By contrast, a region with expression only slightly higher than its surroundings will die shortly after it is born. The ‘lifetime’ of a hotspot, the difference between its birth and death threshold, thus measures its significance - how clearly it stands out from the surrounding tissue.

This information is summarised in a *barcode diagram* (figure 1B(iii)), consisting of a bar for each hotspot spanning from its birth to death threshold (Wasserman 2018). The CoSS is computed as the $L^2$-norm of the barcode (figure 1B(iv)), i.e. by summing up the squared lengths of each bar and taking the square root (Carrière et al. 2015; Adams et al. 2017). The CoSS thus incorporates both the number of distinct spatial features in a gene’s expression as well as how prominent each feature is. A gene with multiple well-defined expression hotspots will have a high CoSS, whilst a gene with a highly diffuse or uniform expression will have a low CoSS. This metric can then be used for downstream tasks that involve comparing the spatial expression structure of different genes.

Biologically, a high CoSS indicates that a gene’s expression is organised into distinct spatial domains, i.e. contiguous regions of high or low expression. This may reflect underlying tissue architecture, pathological processes such as fibrosis, or functional compartmentalisation. Conversely, a low CoSS is indicative of spatially diffuse expression without clear spatial organisation, which may reflect housekeeping genes expressed uniformly across tissue, or a pathological breakdown of normal tissue structure. Importantly, the CoSS quantifies spatial organisation of expression, a geometric feature of a gene’s activity, distinct though not necessarily independent of cell identity or gene function.

### Spatially Variable Gene Identification

Once a CoSS has been computed for each gene, SVG identification can be done by declaring all genes with a CoSS above some threshold to be spatially variable. A threshold can be automatically selected for a given sample by looking for a point of maximum curvature in the CoSS-Rank plot, where genes are ranked by their CoSS (figure 1D), but if desired any practitioner-selected cutoff can be used. Indeed, this flexibility in how permissive one wishes to be about what level of spatial structure counts as ‘spatially variable’ is one of the advantages of the continuous spatial structure score approach to SVG identification.

## Methods

In this section we fill in the technical details omitted in *Method Overview*. The reader who wishes to see first the capabilities of the CoSS score when applied to real data may skip this section and proceed straight to *Results*.

Mathematically, the output of a spatial transcriptomics experiment can be modelled as a collection of weighted point clouds, one for each gene. The data for a single sample consists of the co-ordinates $(x_i, y_i)_{i=1:n_\text {wells}}$ of each well, and for each gene *g* a sequence of weights $(w_i)_{i=1:n_{\text {wells}}}$, where $w_i$ is the expression of *g* in well *i*. Here we restrict our attention to well-based spatial transcriptomics data, in which the wells lie on a regular hexagonal or square network structure (supplementary figure 2A,B). See *Supplementary Methods* for how we automatically align the given well co-ordinates to a network structure.

To each of these weighted point clouds we wish to associate a number measuring the amount of spatial structure therein. As described in *Method Overview*, we do this by computing the 0-dimensional persistent homology of the upper star filtration of each weighted point cloud, then taking the $L^2$ norm of the resulting barcode.

Whilst the underlying idea is straight-forward, there are a number of features of the problem that necessitate some more sophisticated modifications to this pipeline.

**Fig. 2:**
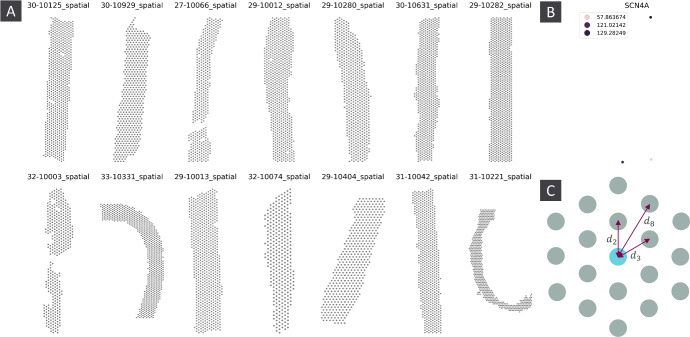
A) Unweighted point clouds showing the location of wells for each of the kpmp samples. B) Example of a gene with low read depth, with expression only detected in two wells. C) Distances from a node to its $2^{nd}$, $3^{rd}$ and $8^{th}$ nearest neighbours in a hexagonal grid

Most significantly, the tissue slices themselves often have non-trivial spatial structure (figure 2A) , and without correcting for this our quantification is liable to be sensitive to this. We want to ensure our methodology is robust to variations in tissue morphology, both to ensure that we are detecting genuine structure in gene expression, and to enable our metric to be used for comparison of spatial structure between different tissue samples.

Additionally, modern well based spatial transcriptomics experiments typically output data for tens of thousands of genes. This means that our methodology will need to be computationally light, and produce a spatial structure score that can be used “as is", without any detailed inspection of the persistent homology outputs.

Finally, as mentioned above, we need our score to be robust to reasonable amounts of noise.

### Smoothed Expression

Most of these issues can be dealt with by applying a suitable smoothing function to each weighted point cloud. Most obviously, applying a smoothing function increases robustness to small amounts of noise and trivial variations in expression from well to well. By picking a suitable smoothing function, we are also able to build in robustness to variations in tissue morphology.

The smoothing function we use is a modified form of the distance to measure of a point cloud (Wasserman 2018). For a probability density $\rho$ on $\mathbb {R}^2$, the distance to measure of a point $p \in \mathbb {R}^2$ is defined in Chazal et al. (2011) as

$$\begin{aligned} {{\,\textrm{dtm}\,}}(p;m) = \frac{1}{m} \int _0^m \delta _a^2(x) \textrm{d}a \end{aligned}$$

for some pre-defined $m \in (0,1)$, where $\delta _a(x) = \inf \{r>0: \mathbb {P}_{\rho }(B(x,r)) > a\}$ is the minimal radius of a ball around *x* covering at least *a* of the mass of $\rho$.

In Chazal et al. (2011) it is shown that if $\rho$ is the empirical density of a point cloud $(x_i)_{i=1:n} \subseteq \mathbb {R}^2$, the distance to measure is given by

1 $$\begin{aligned} {{\,\textrm{dtm}\,}}(p;m) = \frac{1}{k} \sum _{i=1}^k ||p - x^{(i)}||^2 \end{aligned}$$

where $k = \lfloor mn \rfloor$, and $x^{(i)}$ is the $i^{th}$ nearest point to *p* (Wasserman 2018) (we will be evaluating dtm at each well, so $p = x_i$, $x^{(1)} = p$, and $||p - x^{(1)}|| = 0$). This is the average squared distance from *p* to its *k* nearest neighbours, where *k* is minimal such that the combined mass on the neighbours is at least *m*. Note that the definition of ${{\,\textrm{dtm}\,}}(p;m)$ depends on the co-ordinates of all points in the point cloud, but we suppress this in the notation.

The distance to measure preserves many desirable features of a classic density estimator, but is more robust to noise in the input data, and has been observed to be more robust when used as an input to persistent homology (Wasserman 2018). Additionally, by using distance to measure instead of a standard kernel density estimator, we avoid over-scoring samples such as figure 2B consisting of only a small number of wells with measured expression. These can be fairly common in spatial transcriptomics experiments with low read depth, and it is useful to be able to exclude them from any list of SVGs.

Equation (1) naturally extends to the case of a weighted point cloud. Instead of summing over a constant number of nearest neighbours, we let the upper limit of the sum be $k = \min \left\{ N: \sum _{i=1}^N w^{(i)} \ge m\right\}$, which remains the number of nearest neighbour wells needed to reach a combined mass of *m*, but will now vary depending on *p*.

As is, the described smoothing function is highly sensitive to tissue morphology. For wells near the edge or near holes in the tissue, the sequence of distances $(||p - x^{(i)}||)_i$ will increase more quickly than for wells in the bulk of the tissue, leading to artificially higher values of ${{\,\textrm{dtm}\,}}$ at these points. This is an issue that any smoothing function that smooths by looking at local expression is likely to encounter.

We can control for this by replacing $||p - x^{(i)}||$ with $d_i$, where $d_i$ is the distance from any given vertex to its $i^{\text {th}}$ nearest neighbour vertex in an infinite network of the same type as the data, with $d_1 = 0$ (figure 2C). By using network distances, we treat every well like it is in the bulk of the tissue, effectively re-arranging the wells near *p* needed to reach a mass of *m* such that they surround *p* in a regular network structure, and computing ${{\,\textrm{dtm}\,}}(m;p)$ as if this were what the actual data looked like.

Thus the smoothing function we end up using is given by

2 $$\begin{aligned} {{\,\textrm{dtm}\,}}(p;m) = \frac{1}{k} \sum _{i=1}^k d_i^2 \end{aligned}$$

where $$k = \min \left\{ N: \sum _{i=1}^N w^{(i)} \ge m\right\} $$.

Whenever we refer to distance to measure, smoothed expression, or ${{\,\textrm{dtm}\,}}(p;m)$ below, we mean the quantity defined in (2).

In this definition, the parameter *m* controls the degree to which the distance to measure smooths out the original expression data. A sufficiently high value of *m* is needed to maintain robustness to noise and low read depth, but too high a value of *m* may lead to the ‘smoothing out’ of spatial features of interest. For data of the resolution of the spatial transcriptomics data analysed in this paper (a well size of $10 - 55\mu$m), we consistently found that $m = 0.1$ was an effective choice. However, for the much higher resolution data analysed in *Applications to Spatial Metabolomics Data*, we found that $m = 0.1$ led to some over-smoothing of the data, and reduced *m* to 0.01, following a useful heuristic of setting *m* to be the approximately the percentage of the tissue one would expect the smallest spatial feature of interest to take up.

As mentioned previously, we use a distance to measure based smoothing function due to its observed robustness as an input to persistent homology, and as it effectively handles genes with low read depth. Empirically, we find equation (2) to be highly effective, but we have not conducted a comprehensive review of the optimal choice of smoothing function, and whether there may be data configurations for which alternative smoothing functions are more effective. One notable disadvantage of the distance to measure (or any local density based smoothing function) is that it will return artificially higher or lower values near edges in the tissue, a bias we remedy by using network distances as outlined above.

### Computation of CoSS Values

This section assumes a basic understanding of persistent homology. For a brief introduction to persistent homology, see Otter et al. (2017).

We now have a collection of wells with co-ordinates $(x_i)_i$ and smoothed expression values ${{\,\textrm{dtm}\,}}(x_i;m)$, producing a smoothed surface plot of the gene’s expression. The smoothed expression is currently lower in regions of higher original expression, so we first invert this surface about its median, replacing ${{\,\textrm{dtm}\,}}(x_i;m)$ with $z_i = \max \{{{\,\textrm{dtm}\,}}(x_j;m)\}_{j= 1:n} - {{\,\textrm{dtm}\,}}(x_i;m)$. This is to fit our intuition that the smoothed expression should be higher where the original expression is higher, and to integrate better with pre-existing persistent homology workflows.

From this surface, we construct a filtered simplicial complex (*S*, *f*). The nodes of *S* are the wells [*i*], and the edges are the [*i*, *j*] where wells *i* and *j* are adjacent. The index for [*i*] is $z_i$, and the index for [*i*, *j*] is $\min \{z_i, z_j\}$.

The CoSS is computed from the *upper star filtration* on *S*, which is a complement to the standard Vietoris-Rips filtration. The Vietoris-Rips complex of a point cloud for a given radius $r > 0$ is the set of points within a distance *r* of a point in the point cloud, and the Vietoris-Rips filtration is the nested collection of such complexes as *r* increases. By contrast, the upper star complex of a surface over $\mathbb {R}^2$ for a given threshold $z > 0$ is the region of $\mathbb {R}^2$ where the surface is higher than *z*, and the upper star filtration is the nested collection of such complexes as *z* decreases.

Let *H* be the 0 dimensional persistent homology of the upper star filtration on *S*. The *Coefficient of Spatial Structure* (CoSS) is computed as the $L^p$ norm of the barcode of *H*. By default $p = 2$, but this can be altered by the user. A higher *p* biases the CoSS to genes with a smaller number of regions with expression much higher than that of the surrounding tissue.

We also compute a *ratio* statistic, namely the ratio of the $L^{\infty }$ to the $L^0$ norm of the barcode of *H*. This measures how much of the spatial structure in a gene’s expression may be explained by a single feature. A sufficiently high value may be indicative of technical artifacts, (*supplementary methods*, supplementary figure 2C,D).

### SVG Calling

We now have a continuous measure of the spatial structure of the observed expression of each gene. The developed measure of spatial heterogeneity is a continuous quantity, but for many tasks it is useful to have a binary yes/no call for whether a gene is spatially variable.

We compute this by ranking all genes from highest to lowest CoSS, and looking for an “elbow point" in the plot of CoSS versus rank for each gene (figure 1D). All genes with rank below this cutoff *K* are declared as SV.

We compute *K* by looking for the point of maximal curvature in the CoSS-rank plot, using an implementation of the *kneedle* algorithm (Arvai 2023; Satopaa et al. 2011).

## Results

We evaluate the capabilities of the CoSS for detecting spatial structure in gene expression on two public Visium spatial transcriptomics data sets (Lake et al. 2023; Kuppe et al. 2022). These data sets were chosen as they both contain multiple samples of varying, well-defined disease phenotypes, and within each data set all samples were collected using the same data generation protocols. The two data sets also represent two ends of the spatial transcriptomics spectrum with respect to data complexity; Lake et al. (2023) contains samples with highly variant and quite complex morphology, where expression was measured over a small number of relatively large wells, while Kuppe et al. (2022) mostly contains samples with comparatively simple tissue morphology, with gene expression resolved over a much larger number of smaller (though still supracellular) wells .

For the results in this section we provide some biological interpretation, but our main focus is on the ability of our persistent homology pipeline to automatically detect patterns of spatial structure in large data sets. We are principally interested in a) what forms of spatial structure we are able to detect, b) how consistent the results are across samples of varying morphology and size, and c) whether topology exhibits any unique capabilities for spatial structure detection in comparison to other popularly used methodologies. In particular, throughout we compare results obtained using our topology based pipeline to those obtained using SpatialDE, SPARK-X and Sepal. These methods were chosen to represent popularly used SVG identification methods, and the diverse range of methodologies deployed for this task; SpatialDE is based on Gaussian process regression, SPARK-X uses covariance tests, and Sepal deploys mathematical models of diffusion.

### Analysis of Kidney Disease Spatial Transcriptomics Data

We analysed data from the Kidney Precision Medicine Project (KPMP) (Lake et al. 2023). This data consists of 6 Acute Kidney Injury (AKI) and 8 Chronic Kidney Disease (CKD) samples, with expression data on 26027 genes resolved to $55\mu$m wells. The number of wells varies from 317 to 787 across the samples. The tissue samples display distinct morphological variation, including some with highly irregular shapes or with multiple disconnected components (figure 2A). Such variation presents a significant challenge for producing comparable analyses between the different samples.

The number of SVGs identified in each sample based on the automatically selected CoSS-cutoff ranged from 62 to 353. There was no correlation between the number of SVGs identified and the number of wells in the sample (table 1, supplementary figure 3A).

Table 1 provides summary statistics on the number of SVGs identified by each of the comparison methods in each of the kpmp samples, plotted in supplementary figure 3A against the number of wells in each sample. Notably, 1) SpatialDE and SPARK-X, the two methods based on null hypothesis rejection, consistently call more genes as SV than our method or Sepal, the two methods based on continuous quantification of spatial structure, 2) SpatialDE and SPARK-X exhibit much more variability in the number of SVGs called, with SPARK-X exhibiting a substantial correlation between the number of SVGs and the number of wells in a sample, and 3) Sepal consistently identifies less SVGs than we do, for some samples only calling a single digit number of genes as SV.

Spatial feature identification is effectively a form of triage, reducing a large initial number of features down to a smaller number with spatial structure for further analysis. Calling an excessively high number of features as SV increases the downstream burden on the practitioner, whilst calling too few features as SV risks missing out on important biological signal. Topology appears to hit a ‘sweet spot’ with respect to the number of features called as SV, and exhibits greater consistency in the number of features called as SV. Moreover, the use of continuous scores enables a practitioner to triage with greater fidelity the features they wish to analyse further, by varying the score cutoff for a feature to qualify as SV.

**Table 1:** Summary statistics for the number of SVGs called in each of the kpmp samples, for each of the comparison methods. Correlations shown are spearman correlation

			standard	correlation with
method	min	max	deviation	number of wells
Topology	80	332	61.0	-0.11
Sepal	4	93	27.3	0.21
SpatialDE	104	1014	282.9	0.20
SPARK-X	124	3886	1205.8	0.51

#### SVG Examples

To illustrate the range of spatial structures we are able to detect using persistent homology, we exhibit example sets of CoSS identified SVGs, presented in groups with co-localised expression patterns, detected manually using hierarchical clustering on the observed expression values of SVGs.

#### Co-Localised Genes Expressed in the Glomeruli

The type of spatial structure persistent homology is most evidently able to detect consists of multiple distinct regions of high expression surrounded by a background level of lower expression.

**Fig. 3:**
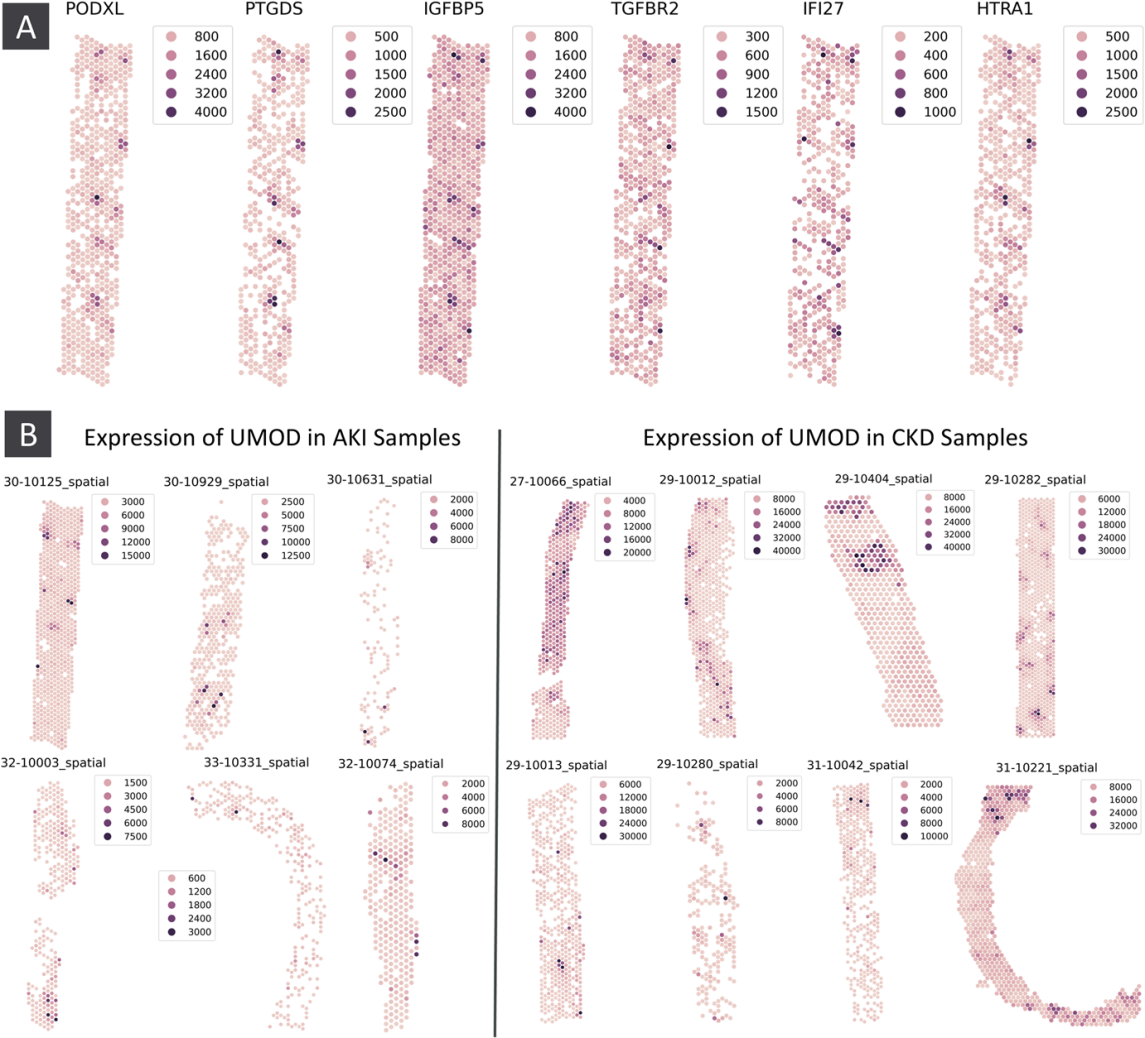
A) Co-expression of PODXL, PTGDS, IGFBP5, TGFBR2, IFI27, HTRA1 in kpmp sample 30-10125 (AKI) at locations corresponding to glomeruli in the tissue. Genes all identified as spatially variable by . B) Expression of UMOD in the AKI and CKD kpmp samples

In one of the AKI samples we identified a group of SVGs highly expressed in regions of the tissue corresponding to glomeruli (figure 3A), as verified by pathologist review of the accompanying H&E images. Some of these, such as PODXL, are well-known glomerular marker genes, whilst others have not been reported as such. In particular, IFI27 is an interferon related gene, indicating the possible presence of immune activity at the glomeruli.

SpatialDE and SPARK-X also identified the genes in figure 3A as spatially variable (except SpatialDE failed to call IGFBP5 as such), but Sepal failed to identify any of these genes as spatially variable.

#### A Single Highly Expressed Well

Another common type of spatial structure in the kpmp data set consists of a single highly expressed well against a background of much lower expression. Whilst such spatial variation is comparatively simple, it is important that such genes are correctly flagged as spatially variable by any automatic SVG identification process.

Supplementary figure 4 shows a collection of CoSS identified SVGs that are all highly expressed in the same well.

The comparator methods struggled here. SpatialDE failed to call COX7B, RNF207 and NOC2L as SV, and SPARK-X, despite calling vastly more genes as SV than us (1161 compared to 188), failed to call RNF207 or NOC2L as SV. Sepal also failed to call any of these genes as SV.

#### CoSS Scores Capture Structural Breakdown in CKD

The continuous quantification of spatial structure provided by the CoSS can be used for additional analysis beyond identifying spatially variable genes.

For example, we can use spatial structure scores to detect differences in the spatial structure of a gene’s expression between sample subgroups. In the kpmp data, we computed for each gene the difference in mean average CoSS between the AKI and CKD samples.

The gene with the highest mean CoSS difference between the AKI and CKD samples was the uromodulin encoding gene UMOD (a marker of kidney tubules). In the AKI samples the expression of UMOD is generally concentrated in a small number of very well-defined regions of high expression, whereas in the CKD samples the expression pattern of UMOD is much more diffuse, with less well-defined regions of high and low expression (figure 3B).

Although they were not originally proposed for this application, we also inspected the mean differences in Sepal scores, and adjusted *p*-values provided by SpatialDE and SPARK-X. In the case of the Sepal score, we consider this analysis a natural extension for continuous measures of spatial structure. In our analysis, UMOD only had the 6163^th^, 6352^th^, and 234^th^ greatest difference according to the Sepal score, SpatialDE q-value and SPARK-X adjusted p-value respectively. Moreover, those genes with the greatest mean difference in each case did not display any consistent notable difference in spatial structure between the AKI and CKD samples (supplementary figure 5).

The reduction in the CoSS of UMOD from the AKI to the CKD samples directly quantifies the structural breakdown that is characteristic of kidney disease progression – what pathologists recognise as tubular atrophy and loss of organised nephron architecture is captured here as reduced topological organisation of a tubular marker gene’s expression. Using persistent homology we can automatically and uniquely detect and quantify the characteristic structural breakdown of chronic kidney disease directly from the spatial transcriptomics data.

#### Overlap Between Different SVG Identification Methods

We investigated the overlap between the SVGs identified by our method, SpatialDE, SPARK-X and Sepal. Table 2 gives the number of genes mutually identified as SV by each pair of methods, in the kpmp sample shown in figure 3A, along with a normalised version of this number divided by the total number of genes called as SV by either method. Our list of SVGs displays some overlap with the comparator methods, whilst identifying plenty of novel SVGs. There is also a substantial amount of difference between the established SVG identification methods, this lack of agreement between SVG identification methods has been observed previously (Charitakis et al. 2023).

The overlap numbers for the other samples are given in supplementary data 1, they do not differ qualitatively from the data for the sample presented here.

**Table 2:** Number of genes mutually identified as spatially variable by different pairs of SVG identification methods in kpmp sample 30-10125. Values in brackets are normalised by the number of genes called as spatially variable by either method. Diagonal entries are the number of SVGs called by each method

	Topology	SpatialDE	Sepal	SPARK-X
	188	58 (0.21)	0	154 (0.13)
SpatialDE	58 (0.21)	144	1 (0.01)	116 (0.10)
Sepal	0	1 (0.01)	69	0
SPARK-X	154 (0.13)	116 (0.10)	0	1157

### Applications to Myocardial Infarction Data

**Fig. 4:**
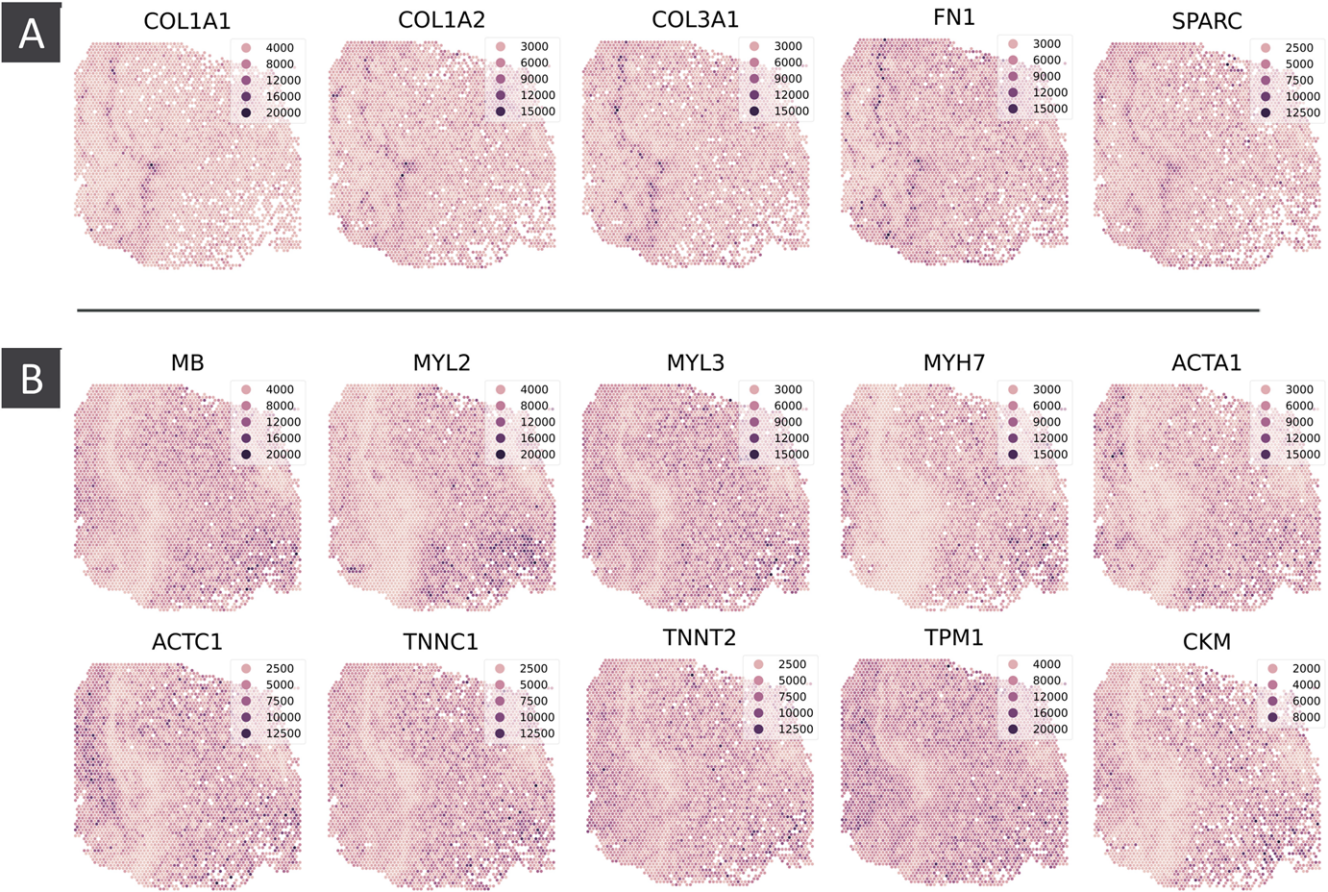
Select SVGs from myocardial infraction sample AKK003-157775 (Ischaemic Zone) - A) Co-expression of COL1A2, COL1A1, COL3A1, FN1 and SPARC. B) Co-expression of MB, MYL2, MYL3, MYH7, ACTA1, ACTC1, TNNC1, TNNT2, TPM1 and CKM. Genes in panel b display a noticeable drop in expression in the same region where the genes in panel a are more highly expressed

To provide a more complete assessment of the capabilities of topology for SVG identification, we also used our methodology to analyse a spatial transcriptomics data set consisting of samples of multiple physiological zones of the heart from myocardial infarction and control patients (Kuppe et al. 2022). These samples are very different from the kidney samples analysed above in that gene expression is resolved to a much larger number of much smaller wells. These samples contain expression on 16272 genes resolved to between 1890 and 4659 $10\mu$m wells, with samples from the borderzone, fibrotic zone, ischaemic zone and remote zone, as well as control samples.

Again we find persistent homology performs favourably compared to the benchmarked methods with respect to consistency in the number of SVGs called, and the correlation between the number of SVGs called and the number of wells in each sample (supplementary table 3, supplementary figure 3B).

#### Spatial Transcriptomics Provides Additional Insight into Cardiac Fibrosis

We identified five SVGs in an ischaemic zone sample all co-localised with COL1A2 (figure 4A). COL1A2 has previously been identified as a driver of cardiac fibrosis (Li et al. 2014), and in Lacraz et al. (2017) the authors use tomo-seq (Junker et al. 2014) to identify a group of genes whose differential regulation correlates with COL1A2 across an infarcted mouse heart. This group includes COL1A1, COL3A1, FN1 and SPARC; here we are able to verify the co-expression of these genes with COL1A2 at finer spatial resolution.

We additionally identified a group of SVGs which display a distinct drop in expression in the same region in which the above genes are highly expressed (figure 4B). By using persistent homology, such “voids" of expression also contribute positively to the CoSS score (Wasserman 2018). Using persistent homology and spatial transcriptomics, we are able to add additional insight into cardiac fibrosis beyond other methods and data modalities.

Sepal failed to identify any of the genes discussed in this section as SV. SPARK-X and SpatialDE identified all these genes as SV, but SpatialDE did so by calling every gene as SV.

### Applications to Spatial Metabolomics Data

Although we developed our pipeline as an application of topology to spatial transcriptomics data, the underlying methodology is agnostic as to the type of measurement recorded at each location. Viewed more broadly, we simply use persistent homology to quantify spatial structure in a weighted point cloud. Beyond spatial transcriptomics, there are many other biomedical data modalities that can be presented in this format. Using topology we avoid making any assumptions about the statistical distribution of gene expression data, so we experimented with applying the pipeline outlined in *Methods* to detect spatial structure in other modalities, making no changes to the underlying methodology.

In this section we analyse a spatial metabolomics (Mass Spectrometery Imaging, MSI) sample (Alexandrov 2023). We find that persistent homology remains effective as an exploratory tool for highlighting metabolites with notable spatial structure, but that whilst our pipeline was robust to differences in the statistical properties of metabolite intensity data compared to gene expression data, the larger size (in terms of the number of points at which metabolite intensity was measured) of the sample we analysed necessitated a different level of smoothing. Additionally, converting the continuous CoSS values into a binary call of spatial variability was complicated by the qualitatively different CoSS-rank profile compared to the spatial transcriptomics samples (figure 1D, supplementary figure 1C). We discuss these points further in *Supplementary Analysis*.

Persistent homology thus shows potential for analysing a broad range of spatially resolved biomedical data modalities, but more work is needed surrounding the auxiliary components of the persistent homology pipeline, and how they may be made robust to differences between data modalities without the need for manual tuning.

#### Spatial Metabolomics Data

In spatial transcriptomics, at each spatial location we measure the *expression* of *genes*. In MSI, we measure the *intensity* of *metabolites*. More specifically, once a grid of pixels on the tissue is decided, within each pixel the molecules of the tissue are ionised, and a mass spectrum is collected. Post data collection, computational software is used to select individual mass-to-charge (m/z) peaks, and the intensity of each m/z peak at each pixel is reported. It is at this point that the data is analogous to spatial transcriptomics data, where each feature has a measurement of abundance at each of a shared set of co-ordinates. We refer to the MSI features by their m/z ratios. The molecular identity of a specific m/z value can be determined by tandem MS (MS/MS) fragmentation, or by matching its intact mass to databases of known molecular masses within a certain mass error range (expressed in parts per million, ppm) (Buchberger et al. 2018).

For the sample we analyse in this section, mass spectrometry imaging was used to measure metabolite intensity on a fresh frozen rat testis at a spatial resolution of $40\mu$m.

#### CoSS Highlights Metabolites Providing Insight into Spermatogenesis

**Fig. 5:**
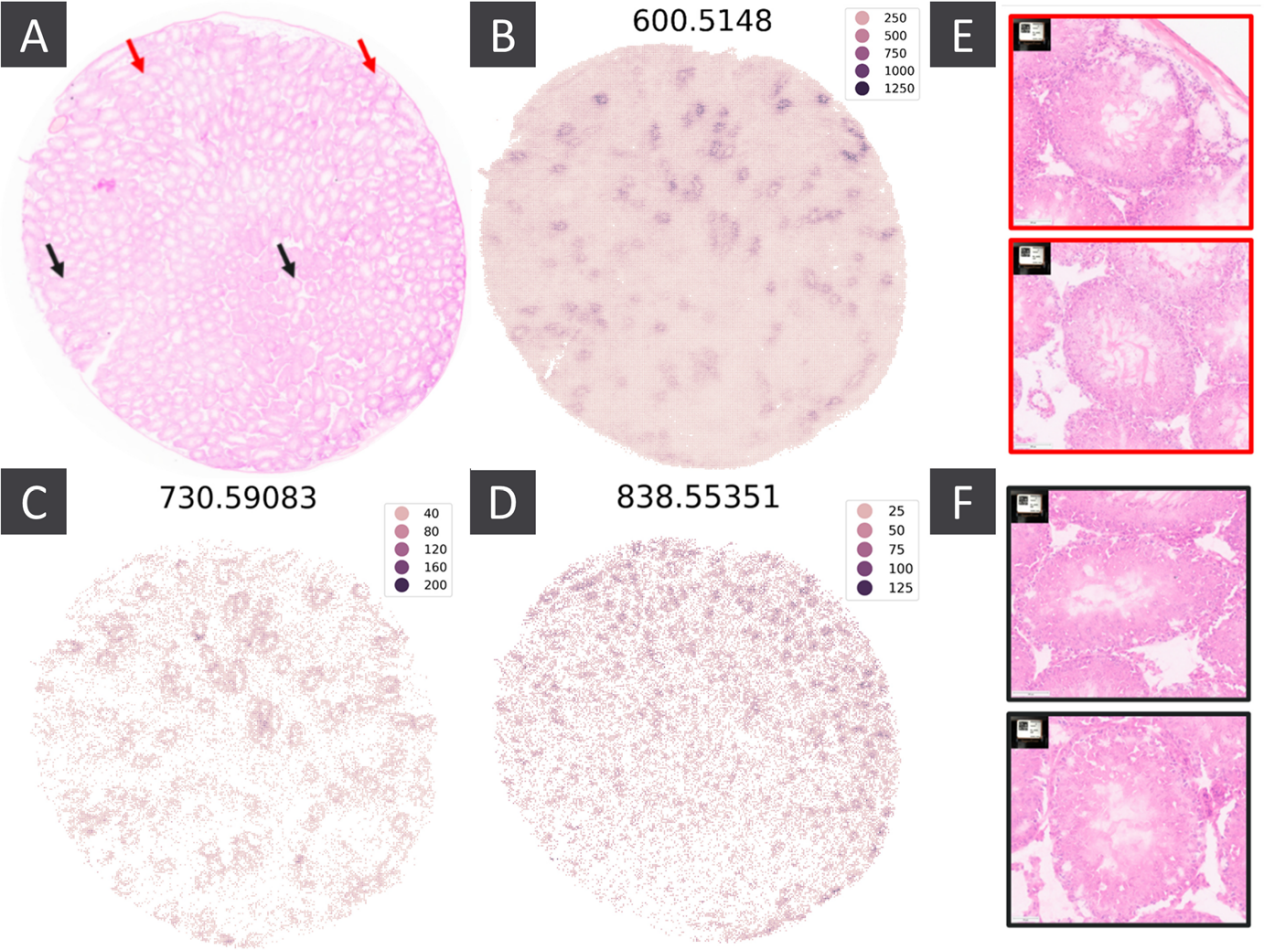
A) H&E slide for the MSI sample. Red and black arrows point to example seminiferous tubules in the early/mid and late stage of spermatogenesis respectively. B,C,D) Spatial intensity of select SVMs. E,F) Zoomed in H&E image for the regions indicated by the red and black arrows in panels A and B

Metabolites 600.5148, 602.5077, and 601.51505 were all flagged as spatially variable, and have co-localised regions of high intensity (figure 5B, supplementary figure 1A). Pathologist review of the accompanying H&E slide (figure 5A,E,F) confirmed that these metabolites have high intensity in regions of the tissue corresponding to seminiferous tubules in the early/mid stage of spermatogenesis. Spermatogenesis occurs continuously and repeatedly in the germinal epithelium of the seminiferous tubules where these metabolites can be found. The m/z features flagged as tubular maturation markers are isotopes from the same molecular species, a ceramide identified as Cer(36:1) and detected as chloride adduct, [M+Cl]- with a mass accuracy of 3.3 ppm compared to theoretical m/z (m/z ratios identified using the Human Metabolite Database (Wishart et al. 2022)). Levels of such sphingolipids, particularly ceramide levels, have been observed changing during the maturation phase of spermatogenesis (Synthase 1997).

Metabolite 730.59083 also shows high intensity in similar regions but is present exclusively round the edges of the tubules (figure 5C), whilst Spatially Variable Metabolites (SVMs) such as 838.55351 displayed hotspots of high intensity exclusively inside the tubules (figure 5D).

Using persistent homology, MSI and histology data, we were able to identify local metabolic perturbations within our sample, and link the m/z features identified with a specific biological process.

## Discussion

We have shown how persistent homology can be used to automatically compute, from spatial omics data on a large number of features, a continuous measure of spatial structure for each feature. We have shown this measure to be a useful exploratory tool for analysing spatial transcriptomics data sets, with unique capabilities in SVG identification and differential spatial expression analysis across multiple biological settings. By computing a spatial structure score based on principled notions of spatial structure, rather than statistical properties of gene expression data, our topology based score detected a broader range of spatial structures and performed more consistently across different biological contexts. Additionally, the underlying methodology was able to produce meaningful results across multiple spatial omics modalities.

Using spatial structure scores, rather than null hypothesis rejection, better reflects the status of “spatial variability" as a continuous descriptive property of biological systems rather than an intrinsic binary biological quantity. Additionally, we have shown that continuous spatial structure scores can be directly useful for multiple analytical tasks beyond feature selection, such as identifying features indicative of changing spatial structures between two sets of samples, an up-to-now manual task largely restricted to pathologists. We expect this to become an increasingly useful analytical tool as typical spatial transcriptomics sample sizes increase, enabling the automatic identification of genes involved in complex disease processes.

Genes with high CoSS and correlated expression patterns can define spatial domains (as with the glomerular marker genes in figure 3A), with hierarchical clustering of SVG expression segmenting tissue into functionally distinct compartments. For more specific spatial questions, such as those regarding local cell-cell interactions, the genes identified by persistent homology can provide an initial feature set to be further analysed by other methods. For example, while the CoSS does not directly quantify cell-cell interactions, it could identify genes marking interaction zones, e.g. immune-tumour interface genes would be expected to have a high CoSS due to expression hotspots at boundary regions, complementing dedicated neighbourhood analysis tools.

Spatial transcriptomics promises to be a powerful complement to digital pathology in assessing patient health. By enabling the rapid triaging of complex data sets, improved SVG selection methods should enable better identification of important features, such fibrotic foci in heart disease or tissue breakdown in chronic diseases. Beyond the examples presented in this paper, a further potential use case would be detecting evidence of tertiary lymphoid structures in cancerous tissue. The presence of such structures has been strongly linked to response to checkpoint therapies (Cabrita et al. 2020; Vanhersecke et al. 2021), and detection of tertiary lymphoid structures from histology images has become an important application of digital pathology, with deep learning approaches enabling automated, reproducible quantification (Li et al. 2023; Rijthoven et al. 2024). Improved identification of genes involved in these spatial-immune features, and a greater ability to distinguish between the degree of immune infiltration between tumours, could serve to improve the allocation of these powerful drugs.

The CoSS is most suited to identifying genes with well defined spatial domains of differential expression at scales larger than the measurement resolution. This measure is particularly effective at detecting patterns of exceptionally high or low expression, and for comparing spatial organisation across samples with variable tissue morphology (see boxout). Persistent homology may be less effective at detecting expression gradients without clear boundaries, or detecting patterns in very sparse expression data. We have only tested the current implementation up to a spatial resolution of 10$\mu$m spot sizes. For higher-resolution platforms such as Visium HD (well size $\approx 2\mu$m), or platforms that detect the location of individual mRNA transcripts, we expect that the principles behind the CoSS should remain applicable, but adjustments may be required to aspects such as the smoothing parameter *m* to capture cell-level rather than multi-cellular organisation, or to the automatic CoSS threshold selection for samples with unusual CoSS distributions, as observed in the spatial metabolomics sample. As the number of points where expression is measured grows, computational bottlenecks may also emerge.

As far as the authors are aware, our work is a novel application of persistent homology to produce an automatic method for analysing big data, that requires no knowledge of persistent homology to be used by a practitioner. That a simple “out of the box" application of persistent homology produced meaningful biological results for multiple spatial omics modalities indicates the broad potential of TDA for analysing complex spatial biological data.

This work highlights persistent homology as a promising tool for the automatic comparison of spatial patterns in gene expression, and in particular for the identification of spatially variable genes, with unique advantages over other commonly used methodologies in its consistency and ability to detect a broad range of spatial structures. Looking forward, a more thorough empirical and theoretical evaluation of the capabilities of persistent homology for SVG identification, a more complete analysis of the effect of different choices for auxiliary parts of the topology pipeline, such as the smoothing function, and further investigation of the use of the CoSS score for other analytical tasks would be valuable for the establishment of persistent homology as a standard out-of-the-box tool for analysing spatial omics data sets.

Nevertheless, persistent homology remains an effective and easy to use exploratory tool for highlighting patterns in spatial transcriptomics data sets, and appears to have unique advantages compared to other popularly used methodologies with regards to the consistency of results across different biological settings, the ability to detect a much broader range of spatial structures, and to enable a wider range of analytical tasks.
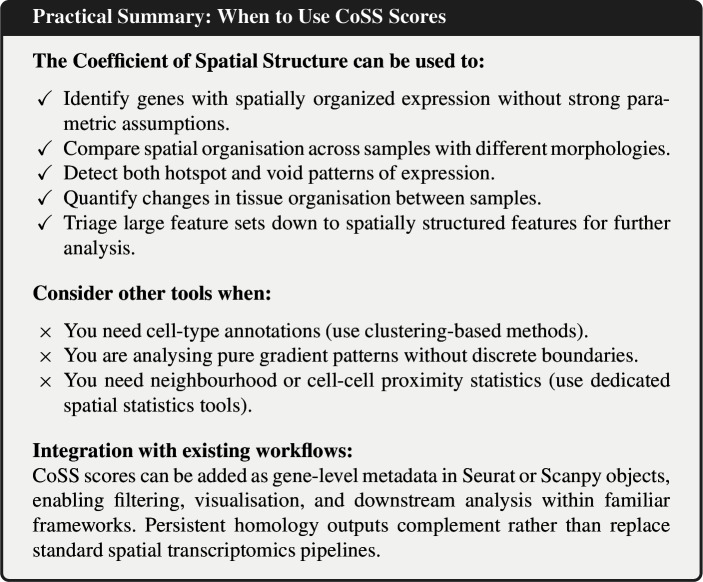


## Supplementary Information

Below is the link to the electronic supplementary material.

Supplementary file 1 (pdf 5072 KB)

## Data Availability

The kidney disease and myocardial infarction data sets are both publicly available at https://www.kpmp.org/available-data and https://zenodo.org/records/6578047. For more details, see Lake et al. (2023) and Kuppe et al. (2022) respectively. The mass spectrometry data may be obtained in accordance with AstraZeneca’s data sharing policy described at https://astrazenecagrouptrials.pharmacm.com/ST/Submission/Disclosure. Requests to access the data described in the current article can be submitted through https://vivli.org/members/enquiries-about-studies-not-listed-on-the-vivli-platform/.

## Code Availability

The code used for the persistent homology computations in this paper is available under an Apache 2.0 license at https://github.com/Jamie-hb/persist. Persistent homology computations were done using the python package Dionysus (https://mrzv.org/software/dionysus2/).
