# Supplementary material for: Topological Data Analysis for Unsupervised Feature Selection in Large Scale Spatial Omics Data Sets
Source: Bull Math Biol. 2026 Mar 4;88(4):52. doi: 10.1007/s11538-026-01618-2 (PMC12960454; doi:10.1007/s11538-026-01618-2)
Supplement: Supplementary file 1 — (pdf 5072 KB) [file 11538_2026_1618_MOESM1_ESM.pdf]

Supplementary Material

In this document we provide further methodological details, discussion and visualisations designed to supplement the main text. The material here is designed to be read in conjunction with the main paper, and we use the same terminology and notation as defined therein.

Supplementary Analysis

Applications to MSI Data

Some differences in the MSI sample compared to the spatial transcriptomics samples necessitated some modifications in the application of persistent homology to identify spatially variable metabolites. These modifications highlight both some of the finer details of our pipeline, as well as some differences between spatial transcriptomics and MSI data. This exhibits some of the difficulties that can arise when translating a data analysis methodology from one spatial modality to another.

Larger Sample Size Necessitates a Different Level of Smoothing

Recall that we initially apply a smoothing function to the raw expression (or in this case intensity) data, with a parameter $m \in (0, 1)$ controlling the level of smoothing. As m increases, the percentage of the tissue a spatial feature must take up in order to not be completely smoothed out increases. For all the spatial transcriptomics samples we analysed, we found that $m = 0.1$ struck a good balance between smoothing out noise, whilst retaining spatial features of interest. The MSI sample we analysed contained a significantly larger number of points where the intensity was measured, and we found that a lower value of $m = 0.01$ struck the appropriate balance.

In general, the lower the proportion of the tissue sample one might expect a spatial feature to take up, the lower one should set m. This will depend on both the number of points and the spatial resolution of the sample.

SVM Calling Complicated by Different CoSS-Rank Profile

For the spatial transcriptomics data sets we analysed, the vast majority of genes had very little spatial structure (and thus a very low CoSS), and the CoSS-rank plot possessed a very sharp elbow that enabled a sensible cutoff to be decided (figure 1d, supplementary figure 1c). For the MSI sample the CoSS-rank plot showed a gradual decline in the CoSS before a sharp drop off at the end (supplementary figure 1c). This complicated the process of making a binary call of spatial variability.

One possibility is that this CoSS-rank profile was driven by technical artefacts that affected a large proportion of the data set. For example, we observed that a large proportion of the metabolites displayed higher intensity and/or more spatial variation towards the top of the tissue sample (supplementary figure 1b). This effectively adds some spatial structure to all affected metabolites, but produces greater CoSS inflation for those affected metabolites with otherwise low spatial structure,

blunting the sharp elbow structure we see in the CoSS-rank plots for the spatial transcriptomics samples.

Alternatively, there may generally be a more gradual gradient of spatial structure in MSI datasets, lessening the distinction between spatially variable and not when making a binary call. Further work is needed on analysis with continuous spatial structure scores in the absence of a sharp 'elbow'.

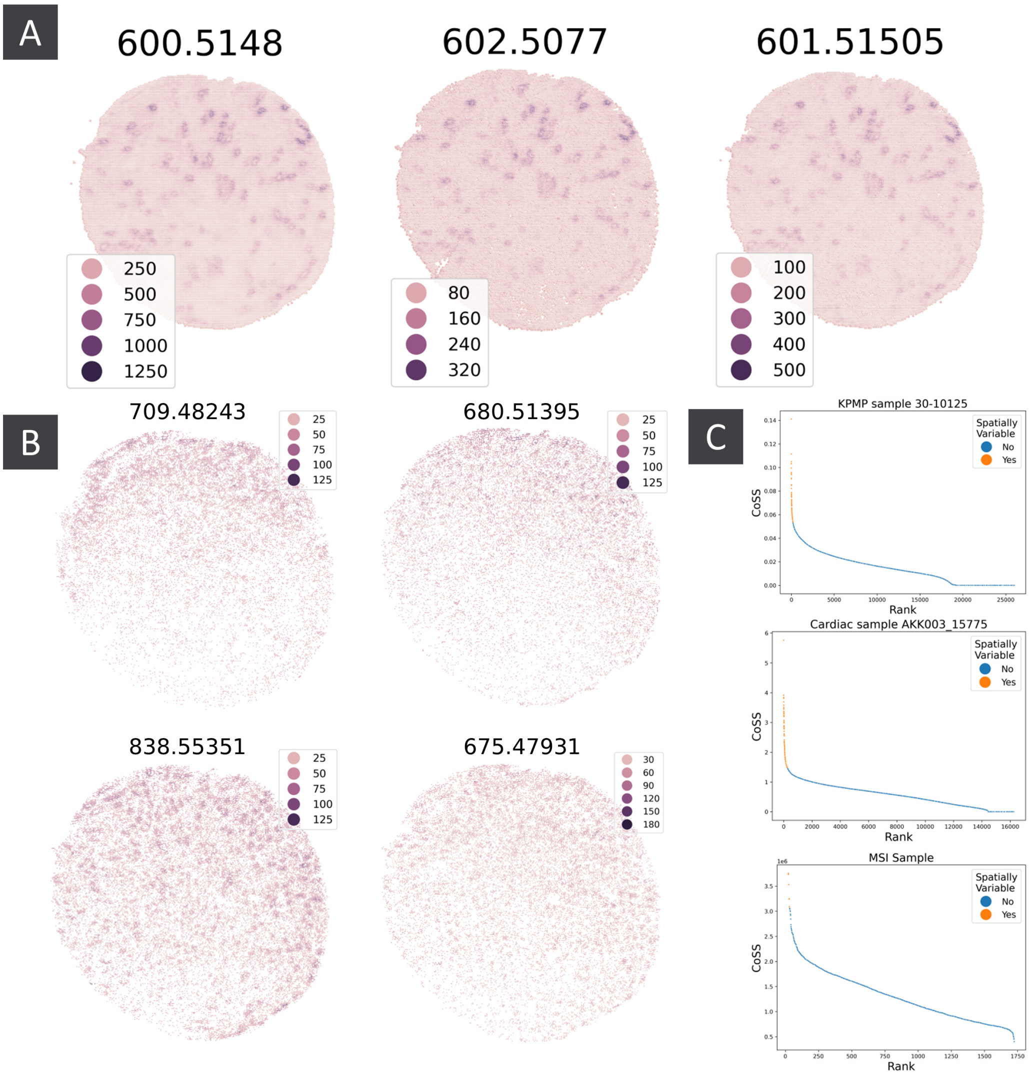

Supp Fig. 1: a) Co-localised metabolites of the same molecular species - a ceramide identified as Cer(36:1). b) Examples of metabolites with intensity biased towards the upper half of the sample. Many metabolites displayed patterns like this, and it is unclear whether this reflects genuine biology or sampling artefacts. c) CoSS-Rank plots for exemplar spatial transcriptomics samples from the kpmp and cardiac cohorts, and the MSI sample. The MSI plot displays a heavier "tail", with a large proportion of metabolites displaying some spatial structure.

Supplementary Methods

Here we give some further specifics on the CoSS computation for each gene.

Aligning Well Co-Ordinates to a Network Structure

Given raw co-ordinate data for each well, we automatically align the wells to a regular network of the type specified.

We determine wells i and j to be adjacent in this network if $d_{ij} < Sd_{\min}$, where $d_{\min} = \min\{d_{ij}\}_{ij}$, and S is a scaling factor dependent on the grid type ($S = (1 + \sqrt{3})/2$ for a hexagonal grid, and $S = (1 + \sqrt{2})/2$ for a square grid). This is equivalent to considering wells to be adjacent if they are separated by a distance less than halfway between the minimal observed distance between wells, and the second nearest neighbour distance in a regular network where the minimum observed inter-well distance is the well separation.(supplementary figure 2a).

We use Sd_{min} as a cutoff rather than just d_{min} to allow for slight mis-specification of the well co-ordinates, or slight misalignment of the wells.

Artefact Detection

Consider the gene expression pattern shown in supplementary figure 2c. Taken as a genuine expression pattern, this is highly spatially heterogeneous. However, it is also possible that such an expression pattern could be the result of technical artefacts. It is typically not possible to determine which is the case from the data alone, so instead PersiST flags such genes as "possible artefacts", and leaves it to the practitioner to decide how to proceed. Here we outline and motivate how this is done.

Such genes are characterised by having the vast majority of their spatial structure explained by a single feature. In the 0 dimensional barcode, this looks like one very long bar against a collection of significantly smaller ones (supplementary figure 2d). Thus to detect possible artefacts, we look at the ratio of the longest bar to the sum of all lengths of bars. In mathematical language, this is the ratio of the L^∞ to the L^0 norm of the barcode.

By default, we flag any gene for which this ratio exceeds 0.9 as a possible artefact. In the authors' experience this strikes the right balance between identifying technical artefacts, but not ruling out genuine patterns of spatial variability.

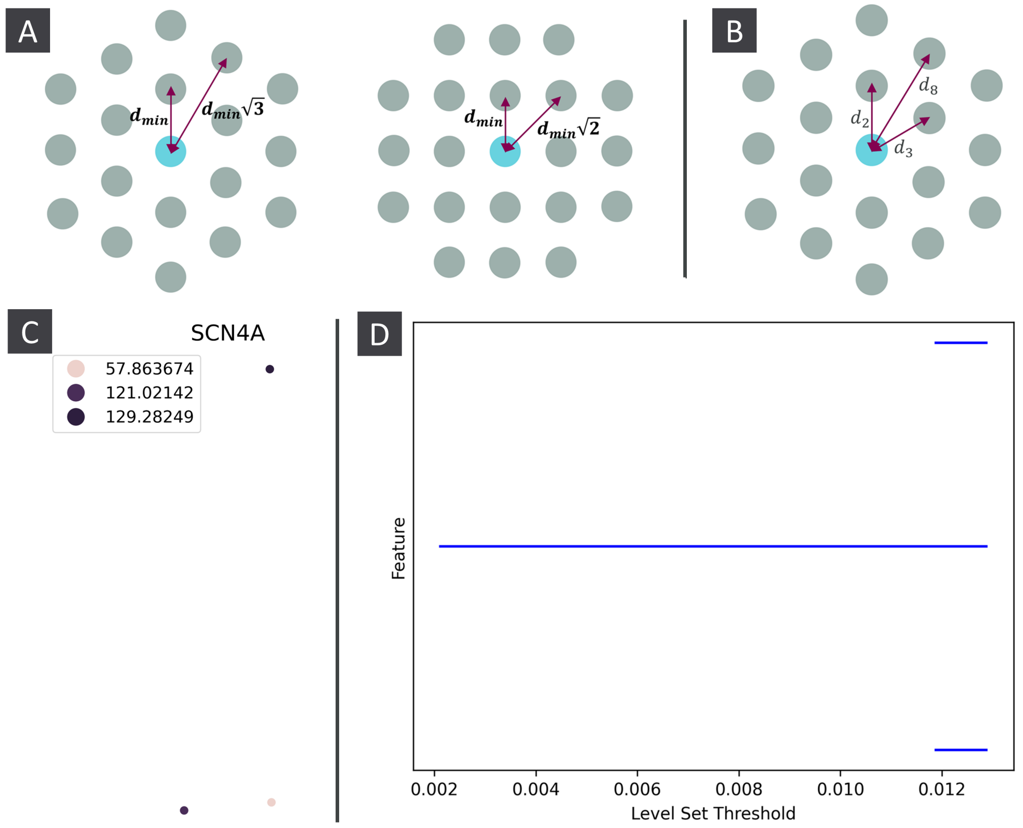

Supp Fig. 2: a) Distances between a central well (light blue) and its nearest and second nearest groups of neighbours, in a hexagonal and square mesh. We consider wells to be adjacent provided that they are separated by less than halfway between the minimum observed inter-well distance and the second nearest neighbour distance in a perfect grid where the minimum observed inter-well distance is the well separation. b) Figure illustrating the 2nd, 3rd, and 8th shortest distances between a central node (light blue) and the surrounding nodes (grey) in a hexagonal grid. $d_1 = 0$, as the distance from the light blue node to itself. c) Expression of SCN4A in kpmp sample 30-10125 and d) the corresponding barcode diagram. The barcode is dominated by a single large bar, with a ratio of 0.91, so this is flagged as a possible artefact.

Supplementary Tables

	CoSS	Sepal	SpatialDE	SPARK-X
CoSS	1	-0.003	0.302	0.159
Sepal	-0.003	1	-0.001	-0.003
SpatialDE	0.302	-0.001	1	0.343
SPARK-X	0.159	-0.003	0.343	1

Supp Table 1: Adjusted Rand Index (ARI) for the different SVG identification methods applied to the kpmp data set [13]. Values shown are mean average ARI across all samples.

	CoSS	Sepal	SpatialDE	SPARK-X
CoSS	1	0	0.25	0.14
Sepal	0	1	0.001	0.0001
SpatialDE	0.58	0.04	1	0.38
SPARK-X	0.55	0.02	0.58	1

Supp Table 2: Overlap percentage for different SVG identification methods on the kpmp data set [13]. The entry in row i column j is the percentage of genes called as SV by method j that method i also calls as SV, averaged across all the samples.

method	minimum	maximum	standard deviation	correlation with number of wells
CoSS	44	351	77.1	0.04
Sepal	0	491	133.1	0.66
SpatialDE	662	15960	4475.0	0.34
SPARK-X	125	3918	1013.8	0.29

Supp Table 3: Summary statistics for the number of SVGs called in each of the myocardial infarction samples [14], for each of the comparison methods. Correlations shown are Spearman correlation.

	CoSS	Sepal	SpatialDE	SPARK-X
CoSS	1	-0.004	0.03	0.2
Sepal	-0.004	1	0.047	-0.008
SpatialDE	0.03	0.047	1	0.117
SPARK-X	0.2	-0.008	0.117	1

Supp Table 4: Adjusted Rand Index (ARI) for the different SVG identification methods applied to the myocardial infarction data set [14]. Values shown are mean average ARI across all samples.

	CoSS	Sepal	SpatialDE	SPARK-X
CoSS	1	0.004	0.04	0.16
Sepal	0.003	1	0.008	0.001
SpatialDE	0.90	0.43	1	0.94
SPARK-X	0.71	0.01	0.19	1

Supp Table 5: Overlap percentage for different SVG identification methods on the myocardial infarction data set [14]. The entry in row i column j is the average percentage of genes called as SV by method j that method i also calls as SV, averaged over all the samples.

Further Supplementary Figures

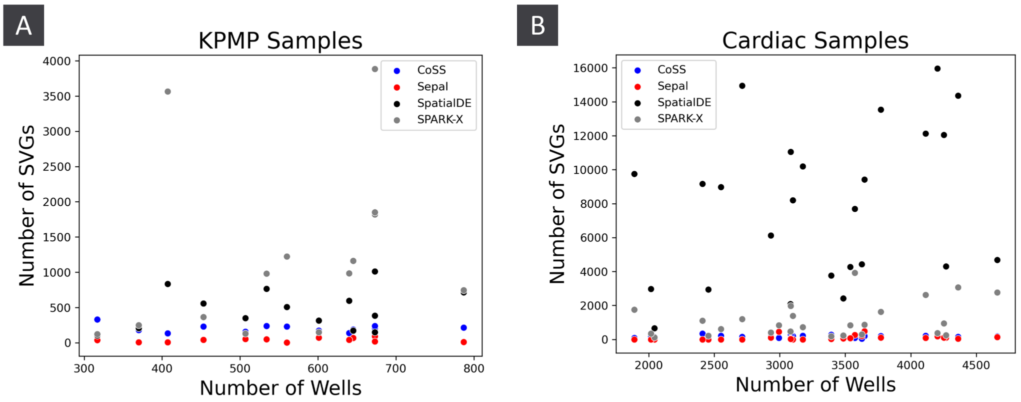

Supp Fig. 3: Plot of the number of SVGs identified by the CoSS, SpatialDE, SPARK-X and Sepal against the number of wells for a) the kpmp samples and b) the cardiac samples.

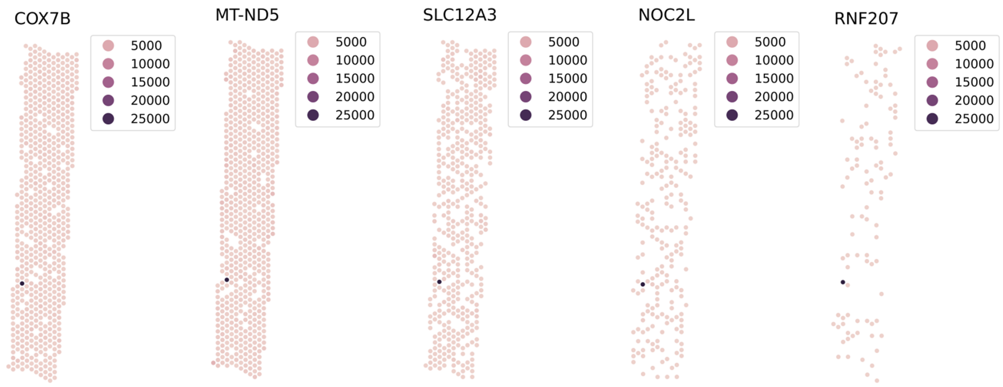

Supp Fig. 4: Co-expression of SVGs COX7B, MT-ND5, SLC12A3, NOC2L, and RNF207 in kpmp sample 30-10135. The expression of all these genes is heavily localised to a single well.

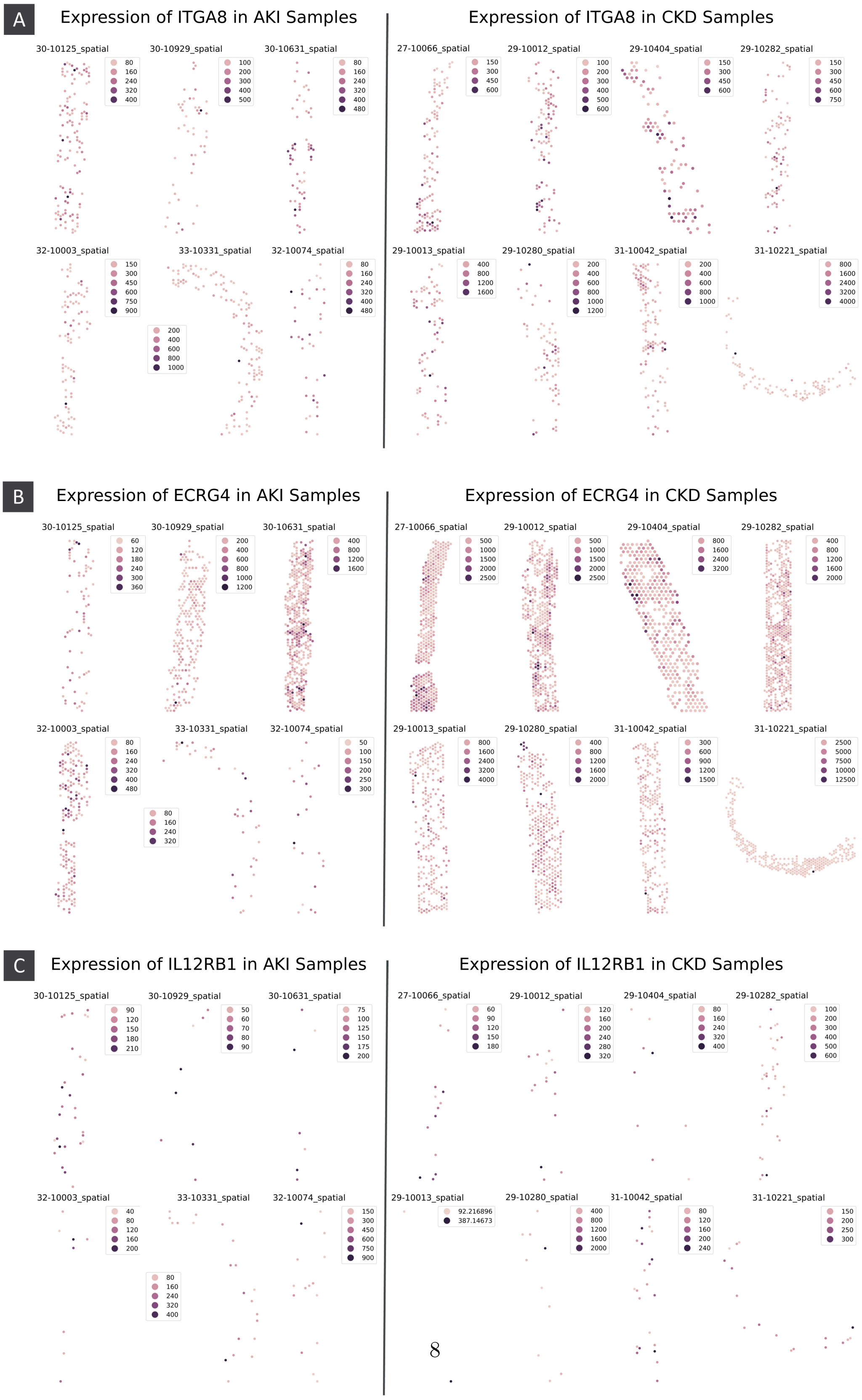

Supp Fig. 5: Expression of the genes with the highest difference in average a) SpatialDE computed adjusted q-value, b) SPARK-X computed q-value and c) sepal score between the AKI and CKD samples in the kpmp cohort.
